# Beyond human-likeness: Socialness is more influential when attributing mental states to robots

**DOI:** 10.1016/j.isci.2024.110070

**Published:** 2024-05-22

**Authors:** Laura E. Jastrzab, Bishakha Chaudhury, Sarah A. Ashley, Kami Koldewyn, Emily S. Cross

**Affiliations:** 1 Institute for Cognitive Neuroscience, School of Human and Behavioural Science, Bangor University, Wales, UK; 2 Institute for Neuroscience and Psychology, School of Psychology, University of Glasgow, Glasgow, UK; 3 Division of Psychiatry, Institute of Mental Health, University College London, London, UK; 4 Chair for Social Brain Sciences, Department of Humanities, Social and Political Sciences, ETHZ, Zürich, Switzerland

**Keywords:** Social interaction, Social sciences, Research methodology social sciences

## Abstract

We sought to replicate and expand previous work showing that the more human-like a robot appears, the more willing people are to attribute mind-like capabilities and socially engage with it. Forty-two participants played games against a human, a humanoid robot, a mechanoid robot, and a computer algorithm while undergoing functional neuroimaging. We confirmed that the more human-like the agent, the more participants attributed a mind to them. However, exploratory analyses revealed that the perceived *socialness* of an agent appeared to be as, if not more, important for mind attribution. Our findings suggest top-down knowledge cues may be equally or possibly more influential than bottom-up stimulus cues when exploring mind attribution in non-human agents. While further work is now required to test this hypothesis directly, these preliminary findings hold important implications for robotic design and to understand and test the flexibility of human social cognition when people engage with artificial agents.

## Highlights

• The more human-like an agent, the more we engage the mentalizing network in the brain
• Perceived socialness was even more influential in engaging the mentalizing network
• Humans still hold a unique advantage over robots during social interactions
• Implications for robotic design and the flexibility of human social cognition

## Graphical abstract

**Figure:**
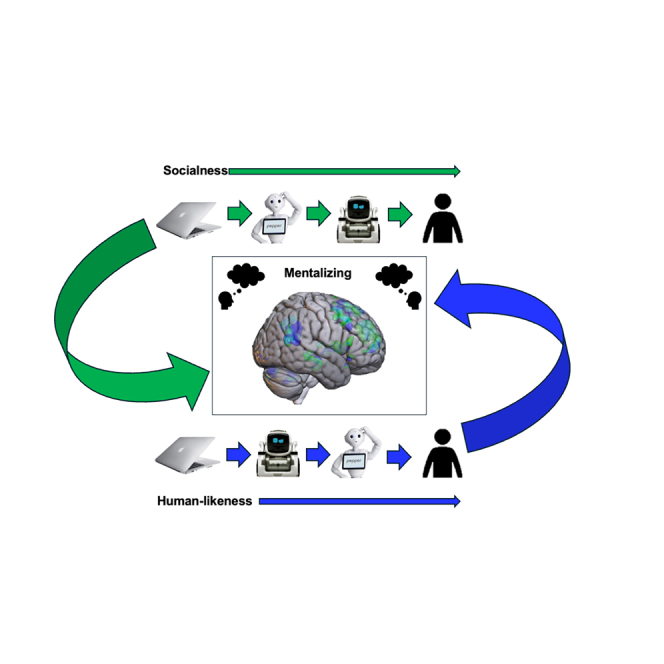

## Introduction

Robots have sparked curiosity and been romanticized in popular culture since von Kempelen’s “Chess Turk” was introduced in 1769. In the mid-20^th^ century, Alan Turing formalized the philosophical debate as to whether “machines think,”^1^ a question that continues to captivate many philosophical and science fiction writers. With the present study, however, we ask what might be thought of as the *opposite* question: namely, regardless of whether robots think, do *we humans* perceive robots as having minds of their own? If so, do we do so primarily based on how human-like the robot looks, or does its perceived socialness also matter?

Robots are already commonplace in assembly lines, factories, and dangerous jobs such as pipeline and fuel tank inspections, as well as underwater and space exploration.^2^^,^^3^ As the deployment of robots in these contexts grows, so does their introduction into more human-centric work, social and leisure domains, for example, assisting with surgeries in healthcare, serving customers in restaurants, supporting children's learning in schools, and supporting older adults who need help with daily living skills (for example,^4^^,^^5^^,^^6^^,^^7^^,^^8^). Robots’ roles in our day-to-day lives so far, however, are typically “single-use” (e.g., robot vacuum cleaners or a robot check-in assistant at a hotel), and the ability of even the most sophisticated social robots to engage us socially is still far removed from depictions in science fiction novels and films.^9^^,^^10^ Rapid advances in hardware and artificial intelligence are expected over the coming decades, making this a crucial time to examine human engagement with robots. This is particularly true in the social domain, if we are to develop machines that can indeed engage and collaborate with humans in complex social contexts.

As adults, humans typically and intuitively think of other humans as having a mind, thoughts, and intentions that are different from their own, a skill known as mentalizing.^11^^,^^12^ Mentalizing is important for social interactions, allowing us to read and react to others’ unspoken mental and emotional states and their intended actions.^11^ Neuroimaging studies have used implicit (e.g., economic games) and explicit (e.g., mind-in-the-eyes) tasks to probe human brain activity associated with mentalizing (for a review, see^13^). This work has identified the so-called mentalizing network, a group of brain regions thought to support thinking about others’ minds. The core regions reliably included as part of the mentalizing network include bilateral temporal-parietal junction (TPJ), medial prefrontal cortex (mPFC), and precuneus (PreC) but additional brain regions, including posterior superior temporal sulcus (pSTS), temporal poles, and posterior cingulate cortex (PCC), have also been implicated.^13^^,^^14^^,^^15^^,^^16^^,^^17^^,^^18^ Briefly, it is thought that the mPFC sits at the top of the mentalizing hierarchy and is the primary source of top-down signals as well as the hub of self-referential processing. The TPJ and pSTS are intermediary in the hierarchy, with the TPJ contributing to metacognitive representations and the pSTS contributing primarily to the processing of social agents and actions (see^14^ for a discussion). The role of the precuneus in the mentalizing system is less clear, also given that a number of other cognitive functions are attributed to it; thus, its functional role is often described as outside of the mentalizing realm (e.g., spatial navigation). Within the mentalizing literature, however, the precuneus is described as potentially belonging at the top of the mentalizing hierarchy (along with mPFC) as a “staging post between implicit and explicit mentalizing.”^14^ For the purposes of the current study, we consider these regions collectively, focusing on engagement of the broader mentalizing system as a whole.

The mentalizing network is readily engaged during interactions with other humans, especially when trying to predict their future actions. Very few neuroimaging studies, however, have directly addressed the extent to which mentalizing brain regions, which have ostensibly evolved to interpret other people’s actions and intentions, also process the actions and intentions of non-human social partners, such as robots. Understanding the extent to which humans mentalize about robots is important for at least two reasons. First, the more we attribute a mind to a robot, the more likely we are to interact with and engage with that robot in a social manner.^19^^,^^20^^,^^21^ Second, examining mentalizing in response to robot social partners tests the flexibility of our social cognitive system by assessing the extent to which a system that evolved to support interactions with fellow humans can also be engaged during interactions with non-human agents (in this case, robots).^22^ Prior neuroimaging studies studying the extent to which humans mentalize about robots have used empathy tasks,^23^^,^^24^ spatial cueing tasks,^21^^,^^25^ and economic games.^26^^,^^27^^,^^28^ Several of these studies demonstrate that human—robot interactions (HRIs) activate the mentalizing network, but to a lesser degree than human—human interactions (HHIs).^26^^,^^27^^,^^29^

One influential theory that might help to explain the pattern of activity reported so far is the “like-me” hypothesis,^30^ which posits that the more human-like a non-human agent is perceived as being, the more readily social brain networks are engaged. Indeed, behavioral data generally support this idea. For example, the more human-like a robot appears, the more a human user will expect that robot to behave like a human.^31^ Furthermore, a robot’s appearance influences our assumptions about its behavioral capabilities^32^^,^^33^^,^^34^ and the extent to which we attribute intentionality or a mind to them.^19^^,^^20^^,^^21^^,^^35^^,^^36^ Likewise, the degree to which we anthropomorphize robots (or attribute human-like qualities to them) has also been found to depend upon a robot’s human-like appearance and behavior.^37^^,^^38^^,^^39^^,^^40^ Given the behavioral evidence, it is perhaps not surprising that similar results are found when examining socio-cognitive brain systems. For example, Krach et al.^27^ reported that the increasing human-likeness of game partners' physical features was associated with increasing engagement of mentalizing network regions during an implicit mentalizing task (in this case, an iterative prisoner’s dilemma game). Together, behavioral and brain imaging findings support the idea that the human-likeness of an interactive partner’s appearance plays a key role in engaging socio-cognitive processes like mentalizing. However, emerging evidence raises the possibility that human-likeness alone may not fully explain which robots are seen as more desirable social partners and, thus, which robot features might be most effective at eliciting the strongest human-like social-cognition processes.^41^^,^^42^ The influence of a robot’s *social* features, per se, on human perception and engagement is an emerging area of research that will benefit from expertise from the HRI, social robotics, and cognitive neuroscience communities.

**Figure 1:**
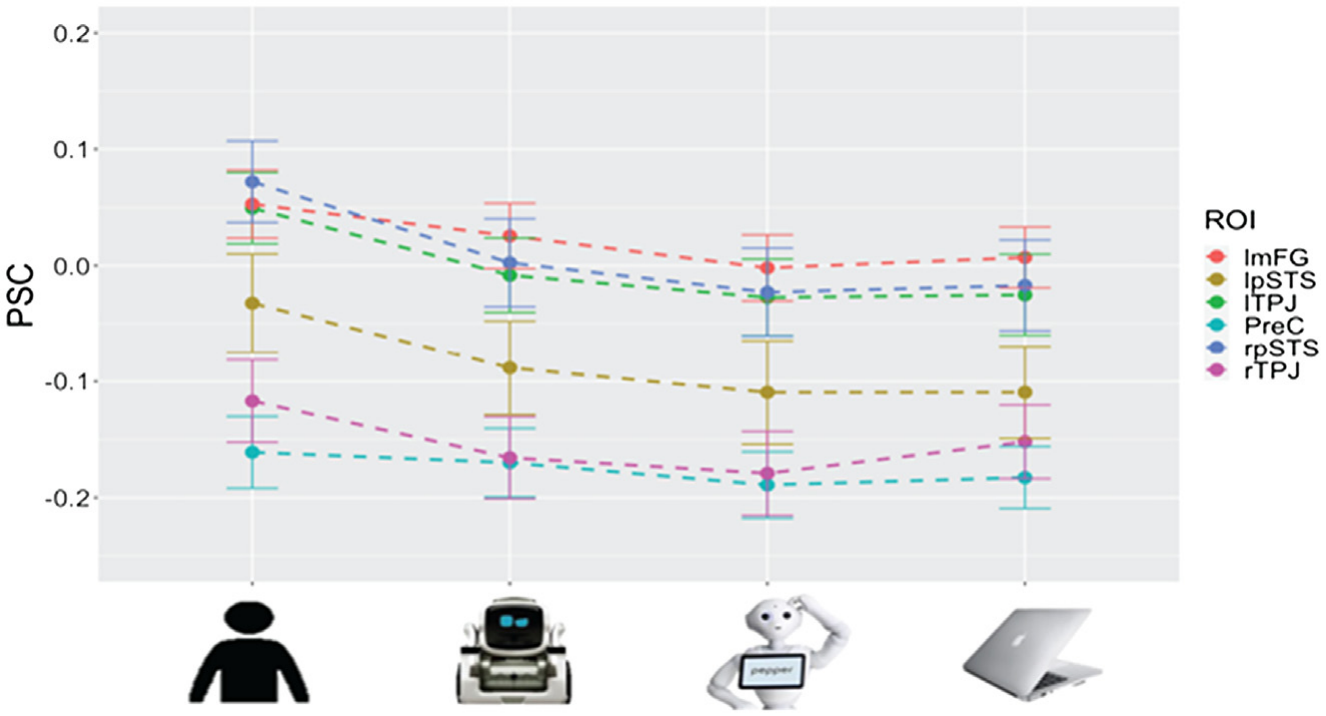
Average percent signal change (PSC) during gameplay in mentalizing ROIs and pSTS with significant within subject rmANOVA (error bars are ±SEM) See also Table S5 & Figure S2.

In the current study, we sought to replicate prior findings that the mentalizing network increases in responsiveness as the appearance of robots increases in human-likeness. In additional exploratory analyses, we sought to explore the extent to which a partner’s perceived *socialness* (independent from human-like physical features) might also contribute to this process. To do so, we used an established implicit mentalizing task where participants play rock-paper-scissors (RPS)^42^ against a human and several artificial agents. We followed an experimental design like that reported by Chaminade et al.^26^ An important feature of our RPS design was that we examined how an individual’s beliefs regarding the nature of the interacting agents are influenced by the human-likeness and socialness of each agent, while tightly controlling all other aspects (i.e., visual, sensorimotor, etc.) of the gameplay interaction. Specifically, participants viewed the same visual stimulus during game play when playing against all four game partners. It was only the videos before and after game play that reminded participants against whom they were playing. This design, therefore, necessitates reliance on top-down knowledge cues regarding the other player to drive neural activation during game play. The RPS game itself is familiar across cultures and age groups, and if it is unfamiliar, it is easy to learn. Also, like Chaminade et al.,^26^ we used videos of game partners to increase the sense of live interactions during game play. We controlled wins and losses across all game partners and explicitly told participants that the robot competitors had been endowed with artificial intelligence and would play strategically. Similar to Krach et al.,^27^ we included two robotic partners that differed in their human-like appearance. One robot appeared humanoid, with clear human-like features including a body, torso, arms, hands, fingers, head, and eyes. The other was a mechanoid robot, which had expressive eyes but no other human-like physical features (refer to Figure 1). Importantly, both the humanoid and mechanoid robots in our study are designed to engage people with socially interactive behaviors.

From prior data, we expected that both robots would engage the mentalizing network, though to a lesser extent than the human game partner. Indeed, we preregistered a prediction that the magnitude of response of core brain regions within the mentalizing network (specifically TPJ, mPFC, and Precuneus) would linearly increase as game partners increased in human-like appearance. We further explored the extent to which participants found each robotic game partner fun, sympathetic, competitive, successful, strategic, intelligent, and competitive. Here, we hypothesized, again based on previous findings,^26^^,^^27^ that these factors would increase with increasing human-likeness. Finally, in an exploratory analysis, to address questions related to participants’ perceptions of the socialness of the different game partners, we reversed the order of the robots (by changing the rank order) in our linear contrast models, allowing us to test the extent to which this “perceived socialness'' might explain differences in the engagement of the mentalizing network across game partners *better* than simply the agents’ physical appearance.

## Results

### Neuroimaging results

#### Socialness and human-likeness influence mentalizing but socialness is more robust

##### Pre-registered

Repeated measures ANOVAs with game partner as a within-subjects factor was significant in several key mentalizing regions of interest (ROIs) during game play (bilateral TPJ and left middle frontal gyrus [lmFG]), as well as bilateral pSTS. All pairwise comparisons in this section were corrected for multiple comparisons (Bonferroni). Follow-up paired sample t tests in bilateral pSTS and lTPJ revealed that this was largely driven by higher activity in response to the human compared to all other conditions, suggesting that these regions are more reliably engaged by human than artificial stimuli (see Table S5). Right TPJ was an exception, in that, although the human significantly differed from both robots, no significant difference between the human and computer was found. No other significant comparisons during gameplay and within these ROIs remained after correcting for multiple comparisons.

Results from the pSTS revealed significant differences between game players while playing the game [rpSTS: F(3, 123) = 12.39, *p* < 0.001, np = 0.23; lpSTS: F(3, 123) = 6.96, *p* < 0.001, np = 0.15], which was unexpected as there were no visual differences during game play across the four conditions.

Contrary to our expectations, mentalizing regions were not activated above baseline during the RPS games. Average activity across the group was close to zero or, indeed, slightly negative across nearly all conditions (refer to Figures 1 and S2; Table S5).

##### Exploratory

Additionally, the pSTS revealed strong significant differences across game partners while participants watched the introductory video of each game partner before playing commencing each game series [rpSTS: F(3, 123) = 29.40, *p* < 0.001, np = 0.42; lpSTS: F(3, 123) = 13.26, *p* < 0.001, np = 0.24]. While none of the other ROIs revealed significant pairwise differences between either robot and the computer, there was a significant difference between MR and CP in rpSTS (and approached significance in lpSTS) during the video preceding gameplay [rpSTS: *p* < 0.001, d = −0.73; lpSTS: *p* = 0.056, d = −0.32; See Table S5].

#### Linear effect of human-likeness in mentalizing ROIs during gameplay

##### Pre-registered

All mentalizing ROIs that revealed a significant within-subject effect of partner (bilateral TPJ, lmFG, and bilateral pSTS) also revealed a significant linear within-subjects contrast effect of human-likeness (HP > HR > MR > CP), as predicted (refer to Table S5).

##### Exploratory

We explored whether changing the rank order of the robots (in the four-element hierarchy) in the within-subject contrasts according to socialness ratings further bolstered the linear effect (HP > MR > HR > CP; refer to Table S5). Results from behavioral ratings suggested that socialness (as assessed by perceived fun, competitiveness, and sympathy, see below) models were improved by reversing the order of the robots. Indeed, across ROIs, the mechanoid robot evoked numerically higher, though often not significantly so, responses than the humanoid robot. Despite the lack of statistically significant differences between the robots in pairwise comparisons, the linear effect of “socialness” resulted in a larger effect size than the “humanness” model, suggesting socialness may be even more important than humanness in mind attribution toward robots, as measured by engagement of brain regions associated with mentalizing.

#### The mechanoid is more similar to the human than the humanoid or computer

##### Pre-registered

No FWE (*p* < 0.05) or uncorrected (*p* < 0.001) clusters survived simple whole brain contrasts between the humanoid or mechanoid and the computer (refer to Table S4). There were no significant clusters during the [Humanoid (HR) > Mechanoid (MR)] but the inverse contrast revealed a significant cluster (k = 313) in nucleus accumbens (MNI: −4, 10, −10). The [Human Partner (HP) > Computer Partner (CP)] contrast resulted in significant mentalizing clusters in bilateral TPJ, mFG, mPFC, precuneus, rpSTS, IFG, nucleus accumbens, and cerebellum.

To assess whether regions outside our pre-selected ROIs might be sensitive to human-likeness, we tested whether any brain regions showed a pattern of activity such that Human Partner (HP) > Humanoid Robot (HR) > Mechanoid Robot (MR) > Computer Partner (CP). This analysis revealed that rTPJ, precuneus, mPFC, bilateral mFG, and nucleus accumbens, all survived the FWE-corrected peak-level threshold.

##### Exploratory

**Figure 2:**
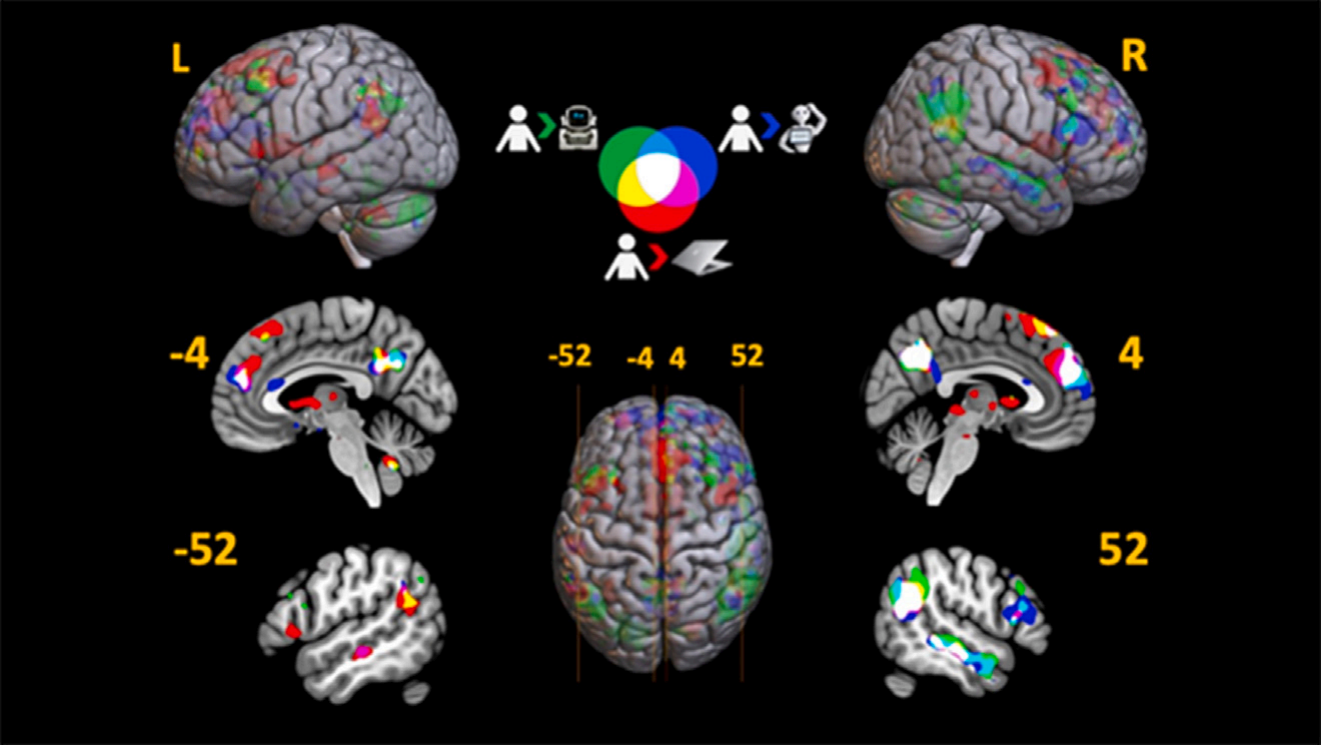
Whole-brain T-map overlap analysis [Human > Computer (Red); Human > Humanoid (Blue); Human > Mechanoid (Green)] See also Table S4 & Figure S1.

When the human was compared to the humanoid and mechanoid robots, several regions associated with mentalizing were significant at the cluster level after FWE correction (refer to Figure 2). The [HP > HR] contrast resulted in significant clusters in bilateral TPJ, precuneus, rmFG, rIFG, and rpSTS after FWE corrections. The [HP > MR] contrast yielded significant engagement of rTPJ, precuneus, rpSTS, and cerebellum after FWE corrections.

In line with our socialness questions, we also tested whether any brain regions showed a pattern of activity if we reversed the order of the robots in our parametric analysis; i.e., so that the order was now: Human Partner (HP) > Mechanoid Robot (MR) > Humanoid Robot (HR) > Computer Partner (CP). Results revealed a similar pattern to both HP > CP and the HP > HR > MR > CP model above but now also included significant clusters in bilateral pSTS, supplementary motor area, rIFG, and lTPJ (refer to Figure S1 and Table S4).

### Behavioral results

#### Manipulation check

During verbal debriefing with participants, 6 out of 42 neuroimaging participants questioned whether the videos were live during our verbal debriefing. Given this, we re-ran all behavioral and neuroimaging analyses with only the “true believers” (see OSF project page for details). Doing so did not change the findings in either degree or direction of significance. Therefore, the analyses are reported with the full sample, including the non-believers.

#### Debrief questions: Mechanoid perceived as more social, but not intelligent, than the humanoid

##### Pre-registered

**Figure 3:**
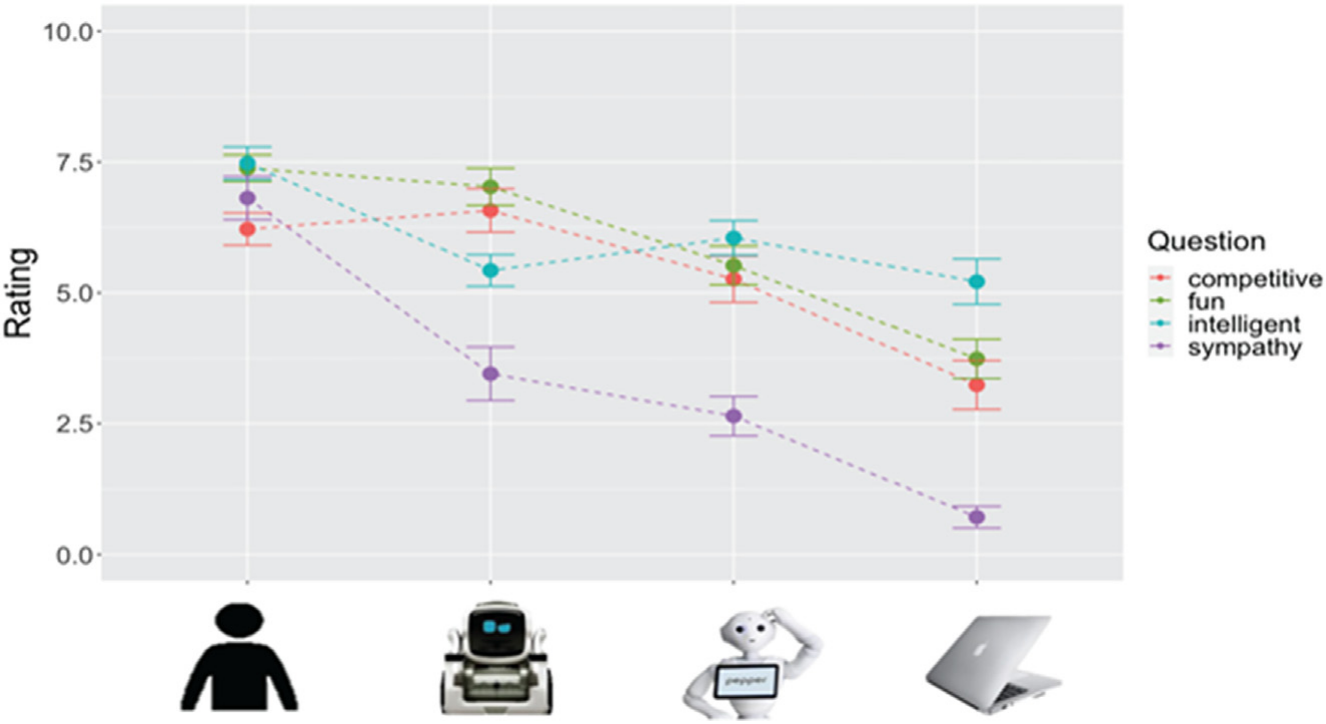
Average Likert (0–10) scale ratings of debrief questions (error bars are ±SEM) See also Table S6 & Figure S3.

All pairwise comparisons in this section were corrected for multiple comparisons (Bonferroni). Greenhouse-Geisser corrections were made if any rmANOVA was found to violate Mauchley’s tests of sphericity (refer to Figures 3 and S3 and Table S6 for details from this section).

We found no effect of perceived *success* in winning [F(3, 123) = 0.50, *p* = 0.685, ηp^2^ = 0.012] or *strategy* employed [F(3, 123) = 0.32, *p* = 0.811, ηp^2^ = 0.008] against each game partner, despite stressing to participants that the computer was using a random algorithm, while the other partners were all trying to win.

*Fun* [F(3, 123) = 33.90, *p* < 0.001, ηp^2^ = 0.453], *Competitiveness* [F(3, 123) = 17.24, *p* < 0.001, ηp^2^ = 0.296], *Sympathy* [F(2.50, 102.58) = 58.59, *p* < 0.001, ηp^2^ = 0.588; Greenhouse-Geisser corrected], and *Intelligence* [F(2.51, 102.91) = 12.16, *p* < 0.001, ηp^2^ = 0.229; Greenhouse-Geisser correction] were all significantly different among the four conditions and followed a significant linear pattern based on human-likeness.

##### Exploratory

However, *Fun*, *Competitiveness*, and *Sympathy* revealed a stronger linear pattern based on socialness, wherein we changed the rank order of the robots in the four-element hierarchy. However, only post-hoc tests on ratings of *Fun* and *Competitiveness* showed differences between robots, where mean ratings for the mechanoid robot were higher than for the humanoid robot (*p* = 0.006 and *p* = 0.049, respectively).

#### Inclusion of others and self: No difference in perception of closeness between the robots or a human stranger

IoS scores varied significantly between the six agents [F(3.70, 148.02) = 122.40, *p* < 0.001, ηp^2^ = 0.754]. Pairwise comparisons of the computer, human game partner, and close friend significantly differed from all other agents and each other on the IoS, even after correcting for multiple comparisons (Bonferroni). Pairwise comparisons of the mechanoid robot, humanoid robot, and human stranger did not significantly differ from each other (please see our OSF page for details).

## Discussion

With the present study, we have replicated and extended previous findings, demonstrating that both human-likeness and perceived “socialness” shape the extent to which participants engage mentalizing regions while playing a competitive game against robotic partners. We found that although human-likeness models showed increased theory-of-mind network engagement (as predicted and pre-registered), the socialness model was even more robust. While this analysis was exploratory and will require replication via hypothesis-confirming follow-up work, it is important for two reasons. First, it suggests that mentalizing processes during interactive exchanges (in this case, a game) are better predicted by how *social* we find our interaction partner, rather than being solely based on how human-like they look. This finding informs how me might update our models of how mentalizing systems can be engaged, particularly by non-human interactants. Secondly, the extent to which humans will ascribe mental states to robots is likely to become increasingly relevant as roboticists develop increasingly sophisticated embodied artificial agents designed to engage human users on a social level. Successful social interactions with such social robots will require people to think about how the robot “thinks.” A better understanding of the factors that influence mentalizing toward and about robots should lead to higher quality and more sustained long-term interactions with robots in social domains (e.g.,^42^).

As with the two previous neuroimaging studies on which we based our current study, we found increasing activation in mentalizing regions with increasing human-likeness.^26^^,^^27^ We also found similar behavioral ratings, showing that while participants did not perceive strategy and success differently across game partners (suggesting participants did not feel that they won or lost more against any one game partners), participants did perceive the game partners differently based on social factors like perceived intelligence, fun, competitiveness, and sympathy. However, unlike previous studies, we explored how these social factors might contribute to mind attribution and found that changing the rank order of robots in the four-element hierarchy in our linear contrast models to reflect participants’ evaluations of socialness resulted in numerically stronger models than those based on the human-likeness of physical features alone.

### Quantifying and exploring human-likeness vs. socialness

While the human and social models were both significant and strong, one possibility for the numerically stronger social model is that the mechanoid robot was perceived as more social because it exhibited higher levels of hedonic factors (as rated by fun, competitiveness, and sympathy) than did the humanoid robot. This finding is consistent with participant qualitative perceptions and behavioral ratings of this same robot in recently published work.^43^^,^^44^ For example, in one scenario from our study, when the mechanoid robot lost the RPS series, its facial expression reflected a pout and it slammed its forklift on the table while moving around in circles in protest. Whereas, when the humanoid robot lost, it responded similarly to the human in a more measured manner, by lowering its arms and shaking its head and/or looking down in defeat. While these differences in personality and behavior were not objectively measured in our study, others report that manipulating social features of robots such as personality,^45^^,^^46^ emotional arousal,^47^ and other hedonic features such as enjoyment and sociability^48^ can increase user engagement, acceptance, and/or satisfaction.

The neuroimaging evidence from this study supports both human-likeness and socialness models when attributing mental states. Bilateral TPJ, bilateral pSTS, and lmFG showed significant increases with human-likeness and a numerically stronger linear increase with socialness. While we expected the whole mentalizing network and pSTS to show a similar response pattern, the exceptions were in mPFC, precuneus, and rmFG.

We were unable to clearly assess the role played by our mPFC, Precuneus, and rmFG ROIs in this study, as we found no significant differences to emerge between the agents during game play. However, a wealth of research has proposed that these regions are central to mentalizing and animacy (e.g.,^13^^,^^16^^,^^18^). As our localizers did not reliably elicit mPFC or rmFG response in this participant cohort, we created ROI from coordinates in the original localizer paper.^49^ It is possible that our “generic” ROIs failed to capture individuals’ peak mentalizing voxels across these regions. However, mPFC and rmFG activation clearly emerges in many of our group whole-brain contrasts. Precuneus clusters in our localizer and main experimental task were large, and the peak cluster from the localizer was more inferior and lateral than the peak clusters in the main experimental task. Last, it is also possible that our localizers produced coordinates for offline social cognition or mentalizing and not for online social cognition.^50^ Thus, mPFC, rmFG, and precuneus may play a role in mentalizing in our study but were perhaps not well captured by our choice of ToM localizer and, thus, the resulting ROI coordinates. Future studies may consider creating simple spheres from t-value peaks reported from our main task experimental data or from peaks reported in other similar papers.

We also explored the response profile of a region in the pSTS that is sensitive to interactive information in observed dyadic social interactions.^51^ This region is nearby, but distinct from, the TPJ, and might plausibly discriminate between game partners. Response in the pSTS discriminated between game partners both during game play and during the video preceding each game series. This was somewhat surprising as the pSTS is largely responsive to the perceptual features of interactions, particularly biological motion.^52^^,^^53^ In our design, there were no social perceptual features to process during game play as players observed the same visual stimuli during game play across all four conditions. This suggests that perhaps top-down knowledge cues may be more influential in this region than previously thought. We further explored these data by testing our linear human-likeness and socialness models on the pSTS data from video 1 and gameplay. Both models were significant, but in this case the social model was numerically stronger during both gameplay (rpSTS only) and video 1 (bilateral pSTS). The pSTS has been implicated as a part of the social cognition and mentalizing networks and has previously been shown to integrate both perceptual and social features.^51^^,^^54^^,^^55^^,^^56^ The pSTS also responds strongly to social interactions between non-human agents such as moving shapes and dots of light that mimic social scenarios (e.g.,^54^^,^^56^^,^^57^) and does so even more strongly when participants are led to believe an object is animate versus inanimate.^36^^,^^58^ One possibility is that because participants were engaging in a real-time interaction in our study, the pSTS was more strongly driven by the social features of game partners rather than their visual features. When motion and visual cues to humanness conflict or are not reliably aligned with more top-down attributions of socialness, the more superior regions in the pSTS may prioritize top-down knowledge cues to humanness in social interactions.

While our neuroimaging and behavioral results indicate a linear effect of human-likeness and socialness across conditions, pairwise comparisons from our ROIs also show that the human partner is perceived significantly differently from all others game partners. While this result is perhaps unsurprising, it suggests that a uniquely human factor still differentiates people from animate non-human entities, even when they are quite “human-like” in appearance or behavior. This result has been reported previously^36^^,^^42^ and is consistent with the idea that the mentalizing system may be best tuned to human actors and human social cues. It is possible with advancing technology and design that the line between robots and humans may blur, and mentalizing regions will become increasingly recruited.

One surprise in our results is that game play did not drive responses in mentalizing regions above baseline. Our expectation, based on prior research,^26^^,^^59^ was that this task would indeed drive engagement of the mentalizing network, at least for the human partner, above baseline. Previous studies^26^^,^^27^ found negative activation to the computer condition and to the non-android robots in mentalizing ROIs, but above-baseline response to the human partner. One possibility here is that our task was particularly demanding, requiring not only mentalizing but also analyzing and remembering strategies for each opponent. It is possible that the negative responses seen in our results are a result of most mentalizing regions being part of, or close to, the default mode network, which tends to deactivate during difficult or demanding tasks.^60^ Additionally, the DMN is also thought to reflect involvement in perceptually decoupled thought processes.^61^ More specifically, our use of a passive rest condition as a baseline could have obscured important changes in activation in response to the task. For example, during minimal baseline tasks (such as passive rest), mind-wandering and other internally generated thoughts (as opposed to those evoked by external stimuli) are likely to occur, and this could comprise similar social cognitive processes at equal or greater magnitude to those required by the more focused task-related processing.^60^^,^^62^ If social processes (e.g., mentalizing) are higher at rest than in the task, then we should see what looks like deactivation when comparing the experimental conditions to the passive baseline. Future studies might consider using an active, rather than passive, baseline^63^ for teasing out the difference in social responses to different partners and computing response differences across those experimental conditions.

It is also possible that the activation in the mentalizing network was attenuated because participants were not actively viewing their game partners during game play and therefore were not receiving a constant stream of visual and social feedback in real time as they would have in “real life.” Instead, perhaps they were relying on memory or impressions of their game partners when playing. Future studies might more robustly activate ToM regions during the game with real-time feedback and/or actual live gameplay. Overall, however, the results are consistent with our pre-registered hypothesis as higher activation levels (or less deactivation) for humans emerged as compared to robots and for robots as compared to the computer condition.

As with previous studies, and unbeknownst to the participants, we controlled wins and losses among game partners so our findings could not be explained by winning or losing more to any one partner. Participants’ ratings of success and strategy against each of the four game partners did not significantly differ, suggesting that they accurately perceived their own performance, including that their strategy did not work any more efficiently for one partner than another, like previous findings.^26^ Therefore, it is unlikely that our findings are due to perceived differences in difficulty in playing each partner. Employing a strategic approach to the game likely relates to thinking about the mind of the other player and thus to activity in the mentalizing network. As a result, participants in this study may have reduced their mentalizing about game partners as they found that their strategies were not working. Future studies might look at manipulating wins and losses or alter initial briefing instructions to create different impressions of each game partner’s fun and competitiveness to explore the extent to which socialness can be manipulated to influence mind attribution toward robots.

### Theoretical implications

Our results support growing evidence emerging from the intersection of social robotics and social neuroscience that multiple routes exist to non-human agents being perceived as “like-me,”^36^^,^^42^ including not only a human-like appearance or motion profile but also being perceived as “social” based on behaviors or background knowledge about a robot. Significant R&D investment continues to fuel the development of socially interactive robots with whom human users can intuitively and effectively collaborate, which often attempt to capture as much human-likeness as possible while also avoiding the uncanny valley.^64^^,^^65^^,^^66^^,^^67^^,^^68^^,^^69^ However, the extent to which an agent is perceived as “like-me” extends beyond physical form, capabilities, and movement, and growing evidence supports that prior knowledge about and the perceived socialness of a robot may more strongly influence their reception (and people’s ability to collaborate or cooperate with them in an intuitive manner) in social settings.^41^^,^^43^^,^^70^^,^^71^^,^^72^^,^^73^^,^^74^^,^^75^

A few neuroimaging studies have investigated how these top-down knowledge cues and bottom-up stimulus cues influence perceptions of animacy and the flexibility of our social cognitive system. One study found that stimulus cues overrode knowledge cues to animacy,^76^ whereas others found the inverse, knowledge, not stimulus, cues more strongly influenced animacy perception.^42^^,^^77^ Yet, a key mentalizing region (rTPJ) was most sensitive when *both* stimulus and knowledge cues to animacy were presented compared to when only one (or none) of those cues were present.^36^ These various findings are likely influenced by the type of task and cues used, and our study adds to the narrative that top-down knowledge-based cues of socialness can be just as, if not more, powerful for driving mind attribution during social interactions with artificial agents than bottom-up visual cues to human-likeness alone.

Therefore, physical features denoting human-likeness may not be the most important consideration for those designing socially engaging robots, and instead a reorientation toward an emphasis on socialness may be more fruitful for fostering social behaviors and attitudes toward robots. Ultimately, our findings set the stage for future work to disentangle not only which physical and social features play the most important roles in mind attribution to artificial agents but also how ongoing experience with such agents changes and develops such perceptions.

### Limitations and future directions

Throughout our study we examined human-likeness and socialness using linear models. However, it is noteworthy that these concepts are frequently regarded as non-linear, especially when applied to social robotics.^78^^,^^79^ Even in our study, results in one of the ROIs (the rTPJ) may have been better explained using a non-linear function. One possibility to explain why our linear models for human-likeness and socialness were still robust in most ROIs is that, while our humanoid had a human shape (with a torso, arms, and head), neither our humanoid nor mechanoid robots approached realistic human-likeness. If we had included more realistic human-looking robots (androids) in the design, non-linear models may have offered a better model fit.^79^ During the experimental design phase in future studies, consideration of which conditions might best test whether linear or curvilinear functions most parsimoniously account for neural activity, and whether which function best fits the data could vary across regions of interest, should be driven by several factors, including robot physical and social features.

Next, while we designed our video stimuli to be as believable as possible, ultimately 6 of our 42 participants did not believe that they were playing a live game. Removing the non-believers from analyses, however, did not change our overall findings (see OSF for more details). Thus, we consider our results to reflect brain response when participants are engaged in true real-time interactions with their game partners. In the past decade, a discussion has emerged around designing real-time social interactions in a genuinely interactive context. This movement is grounded in the understanding that social cognition may be fundamentally different during active versus passive social interactions, termed “second-person neuroscience.”^80^ A growing but comparatively small proportion of fMRI studies have attempted second-person neuroscience in human interactions; even fewer, to date, have attempted work at the intersection of social neuroscience and social robotics. However, in one fMRI study, participants engaged in real-time discussion via a live-feed interface with either a human or a conversational robot.^81^ Their findings revealed increased neural activity during HHI compared to HRI in specific mentalizing regions, most notably the TPJ (but not mPFC) and social motivation regions, including hypothalamus and amygdala. More neuroimaging work to date has deployed technologies such as EEG or fNIRS to examine direct, embodied HRIs (see^9^^,^^22^^,^^82^ for a discussion). For example, in live-interactive paradigms with robots, most people used mechanistic terms to describe robots.^83^ Further, whether someone tends to favor mentalistic or mechanistic explanations for robot behavior can be predicted from resting-state electroencephalogram (EEG) signals before participants engage in describing robot behavior.^84^ These studies highlight the value of a number of different neuroimaging techniques for exploring second-person neuroscience perspectives in the context of HRI. Our comprehension of real-time mechanisms and the outcomes of social engagement with robots hinges on combining these approaches with rigorous and theoretically driven experimental designs.

A further possible limitation in our study is that we used only people who identified as male, and, therefore, we were not able to comment on gendered effects of mentalizing in the context of social interactions with robots. We chose male participants because one aim of the study was to replicate previous designs,^26^^,^^27^ which also used only male participant samples. However, the influence of participant’s gender on mentalizing in the context of social robotics is an area of much needed investigation. The broader literature on gendered effects in mentalizing is mixed^85^^,^^86^ but the prevailing narrative suggests that females have a “female advantage,” across cultures, on many social cognition measures, outperforming males on mentalizing tasks.^87^^,^^88^^,^^89^^,^^90^ Indeed, one study found variations in mPFC activation during a ToM task to be more pronounced in women compared to men.^91^ To further complicate matters, the gender of the human player may also be important. Both previous studies that informed our study design used a male human player; in our study, the human player was female. It is possible that this difference in study design could have influenced participant strategy and possibly neural activation. Indeed, prior work suggests that participants play differently depending on the gender of their game partner.^92^^,^^93^ It will be important to thoughtfully consider gender effects of the participants, human confederates, and perhaps even the perceived gender of the robots when planning future research studies using similar designs.

### Concluding thoughts

Our primary findings confirm previous research that human-likeness plays an important role in the attribution of mind to robots. However, our exploratory analyses suggest that the perceived socialness of a robot also plays an equally, if not more important, role than physical features denoting human-likeness in mind attribution. Incorporating knowledge- or experience-based social cues and features into robots that are designed to engage human users on a social level has the potential to increase user engagement and interest for more lasting and higher quality social engagement with our robotic partners.

## STAR★Methods

### Key resources table

**Table:** 

REAGENT or RESOURCE	SOURCE	IDENTIFIER
**Deposited data**		

fMRI data	Mendeley Data	https://doi.org/10.17632/2x9ykks2xx.1 https://doi.org/10.17632/693ty6chcd.1 https://doi.org/10.17632/c48324drrw.1
Group level whole brain results	This paper; Neurovault	https://identifiers.org/neurovault.collection:17268
Behavioral data, stimuli, and additional analyses	This paper; OSF	https://osf.io/t4apv/
Preregistration	AsPredicted.org	https://aspredicted.org/CBG_ZPG

**Software and algorithms**		

MATLAB 2018a	MathWorks Inc	RRID:SCR_001622
Statistical Parametric Mapping 12 (SPM12)	https://www.fil.ion.ucl.ac.uk/spm/	RRID:SCR_007037
Python 2.7	Python Software Foundation	RRID:SCR_008394
Python 3.5	Python Software Foundation	RRID:SCR_008394
Psychopy	https://www.psychopy.org/	RRID:SCR_006571
Psychtoolbox-3 (PTB-3)	Psychophysics Toolbox	RRID:SCR_002881
R Studio	The R Foundation	RRID:SCR_001905
Code for robot introduction & main experiment	GitHub	https://github.com/chaudhuryB

### Resource availability

#### Lead contact

Further information for resources should be directed to Emily Cross (emily.cross@gess.ethz.ch).

#### Materials availability

Robot videos and example human and computer videos are provided on our OSF page (https://osf.io/t4apv/). See the key resources table for details.

#### Data and code availability

• The de-identified fMRI data have been compressed and deposited across 3 sites at Mendeley data and are publicly available as of the date of publication. See the key resources table for details.
• Code for the robot introduction and main experiment have been deposited on Github and are publicly available as of the date of publication. See the key resources table for details.
• Any additional information required to re-analyze the data reported in this paper is available from the lead contact upon request.

### Experimental model and study participant details

#### Human participants

Due to the availability of scanning resources, participants were recruited from 2 sites: (i) the greater Glasgow area (Scotland, UK); and (ii) the greater Bangor area (Wales, UK). Glasgow participants completed the study at the Centre for Cognitive Neuroimaging (CCNi) at the University of Glasgow, while Bangor participants completed the study at the Bangor Imaging Unit (BIU) at Bangor University.

Twenty right-handed males (mean age = 20.95 years; SD = 1.82; range = 19-26) participated from the greater Bangor area and 24 right-handed males (mean age = 22.45 years; SD = 3.63; range = 18-32) participated from Glasgow. There were no significant differences in either age (t(40) = 1.76,p = .087) or education (U = 206.50, Z = -.391, p = .696) between data collection sites. Only males were recruited, consistent with previous studies in this area,^26^^,^^27^ in order to control for any potential effects of gender on mentalizing.^91^ Two participants withdrew from the study due to claustrophobia (1 subject from each site). The final fMRI participant sample included a total of 42 participants (mean age = 21.74 years; SD = 3.03; range = 18-32).

All participants reported normal or corrected-to-normal vision, no history of neurological or psychiatric disorders, and were right-handed as confirmed on the Edinburgh Handedness Questionnaire^94^; mean = 1.48, sd = .34).

All participants reported low familiarity with robots. Median engagement with robots in daily life (measured from 1 (never) to 7 (daily)) was 2 (IQR 1). Median number of robot-themed movies or TV shows seen was 4 (IQR 1) out of the 14 listed (Riek et al., 2011).^95^

All participants provided written informed consent prior to their involvement and received monetary compensation for study participation (£12/hour). All study procedures were approved by the respective university ethics boards: (i) Bangor University (Approval no. 2019-16639) and (ii) Glasgow University (Approval no. 300180110).

Study site was a significantly different between subjects factor in rmANOVA for rTPJ, rmFG, & Precuneus but when we ran site separately for each of those ROIs, the results did not differ from the combined group or change the outcome; therefore, both sites were kept together in the results reported in this paper. Please see our OSF for details on the results from the separate groups. Further, we ran site as a covariate of no-interest in our model estimation and did not find differences in our whole brain data; therefore, the sites were subsequently analysed and reported together (please see our OSF for more details).

### Method details

#### Experimental design

We designed a Rock-Paper-Scissors (RPS) task similar to a previous study,^25^ and followed a similar briefing procedure.^26^^,^^27^ RPS was chosen for its familiarity across ages and cultures, and ease of rule learning. Previous studies have shown this game to engage mentalizing regions when played against human and non-human partners.^26^^,^^29^^,^^59^

Participants saw videos of their respective game partners before and after each 3-game series (refer to Figure S4). Each video was unique and all participants saw the same set of videos. During the pre-recorded videos, the human and robots reacted emotively to winning and losing a round. For example, the human and humanoid often put their hands up (or the forklift for the mechanoid) in exasperation when losing or happiness when winning. The mechanoid had expressive, pixelated eyes and was capable of moving within a restricted space on the table. Whereas, the humanoid had two lights for eyes that could flash but were not expressive and while the humanoid’s arms, head, and torso could move, it did not move its position on the floor during any interactions with participants. All robot videos, and example human videos, are available on our OSF and Mendeley data (see link in key resources table). The computer condition, which participants were told did not have an algorithm to win, involved a screensaver (Apple iMac ‘Flurry’) for both the pre- and post-game videos.

During the game play, participants saw the same visual input across all 4 conditions, namely a score card across the top of the screen (for win/loss/tie in each series), pictures of the rock paper and scissors, and a countdown from 2 to 0 (refer to Figure S4 for an example). To minimize movement in the scanner, we did not utilize synchrony through a “fist-swing” as players might in real-life, rather participants were instructed to select their RPS choice from a button box on ‘0’ in the coutndown. The button press for rock, paper, and scissors and order of the items on the screen during gameplay were assigned randomly across participants.

In-line with previous designs,^26^^,^^27^^,^^59^ participants were told that they were playing a live game and viewing their game-partners through a live video feed, but in reality, neither the remote practice nor the in-scanner games (described below) were live. All videos were pre-recorded and designed to give the impression of a live game. Wins and losses were controlled across the four conditions so that each participant won 10 rounds and lost 10 rounds against each partner. The order in which participants played partners was pseudo-randomized across four 8-minute functional runs.

To give the impression of a live game, participants met all game partners in person in the “game room” and played one truly live, in-person, round of rock-paper-scissors with each partner. They went to the imaging suite to play a “live” practice round of RPS with their partners via the “video feed”. This practice round served to familiarise participants with the game and practice pushing the buttons to register their answer with the correct timing. Participants played each partner twice in each practice round and could complete up to 3 practice rounds (24 total games) to ensure they understood the game before entering the scanner. All participants demonstrated understanding of the game and button presses by the 3^rd^ practice round.

Participants then completed the fMRI task, playing the same RPS game. Each fMRI run contained 5 rounds with each of the 4 partners (20 rounds per partner across all 4 runs), pseudorandomized across participants. In total, each participant completed four, 8-minute RPS runs. After the scan, participants completed several questionnaires (listed below) on a laptop and were then debriefed. The debriefing unveiled the study deception (that the various game partners were pre-recorded, not live, and that all partners used the same random algorithm and were not independently controlled). Both the practice round and game in the scanner were programmed in Python 3.7 and run from the command line (see key resources table).

#### MRI parameters, pre-processing, & GLM estimation

At both data collection sites (CCNI and BIU), stimuli were projected onto a mirror from a projector located behind the scanner. Responses were recorded with an MRI-compatible keypad.

A dual-echo EPI sequence was used to improve signal-to-noise ratio (SNR) in frontal and temporal regions.^96^ All structural and functional sequence parameters are detailed in Tables S1 and S2.

Data pre-processing was carried out in SPM12 (Wellcome Trust Centre for Neuroimaging, London) implemented in Matlab 2018a (Mathworks, Natick, MA, USA). Pre-processing consisted of standard SPM12 defaults for slice time correction, realignment and re-slicing, co-registration, unified segmentation & normalisation, and smoothing; except for a 6mm FWHM Gaussian smoothing kernel. All analyses were performed in normalized MNI space. Block durations and onsets for each of the 4 experimental conditions during Video 1, the RPS game, and Video 2 were modelled by convolving the hemodynamic response function and with a high pass filter of 128s. Head motion parameters were modelled as nuisance regressors. Functional scans provided whole brain coverage.

#### ROI Creation & analyses

Our choice of ROIs was informed by previous studies^26^^,^^27^; however, ROI placement was based on peak activation from the independent localizers (refer to Figure S2; Table S3). Participants undertook two passive-viewing tasks to help identify brain regions of interest after playing the RPS game.

##### Mentalizing

Localizer 1 was a short-animated film (‘Partly Cloudy’; Pixar Animation Studios, 2009) coded for event type (mentalizing, pain, social, and control). We used the mentalizing > pain contrast to identify ROI coordinates in bilateral TPJ, bilateral mFG, and Precuneus independently from our main experimental task. Neither Medial Prefrontal Cortex (mPFC) nor rmFG activation appeared as expected in Localiser 1, therefore, we used mPFC & rmFG coordinates from the original localiser paper^49^ and created 6mm spheres around those coordinates.

##### Social interaction

To localize pSTS, we employed an established localizer which involves passive viewing of 3 conditions: (i) interacting, (ii) non-interacting, and (iii) scrambled point-light figures.^51^^,^^56^ We used the interaction > scrambled contrast (i.e., two human point light figures interacting vs. scrambled dot motion) to derive our pSTS coordinates independently from our experimental task.

We used a control ROI (V1/BA17) from the WFU PickAtlas^97^ as a form of verification that activity differences seen between conditions during game play was not attributable to non-specific whole brain activation differences. In other words, we would not expect differences between conditions in V1 activity during game play, as participants saw the same set-up across all conditions, and this control ROI allowed us to evaluate this possibility.

Group-constrained, subject specific ROIs were created like the STAR Methods described elsewhere^51^ using an uncorrected height threshold of p < .0001. This protocol creates subject-specific ROIs based on independent data (i.e. localizers). Briefly, we established an initial 6mm bounding sphere centred around the peak T-value from group activation in our pre-registered localizer contrasts (i.e. interacting vs non-Interacting, mentalizing vs pain). Within this initial bounding sphere, we employed a leave one subject out (LOSO) iterative process based on group level analyses, resulting in a more refined search sphere. Finally, we generated subject specific regions of interest (ROIs) within this constrained search space by selecting the top 100 contiguous voxels for each subject, thereby accounting for inter-subject variability within these restricted search spaces. Percent signal change was then extracted from ROIs using in-house scripts in Matlab 2018a and the MarsBar toolbox.

None of the ROIs overlapped. Both right and left TPJ were slightly shifted so the entire sphere was within the boundaries of the brain; all other ROIs created from the localisers remained true to the peak activation. Please refer to Supplementary Figures for all ROI coordinates.

#### Behavioral measures

##### Debrief questions

###### *Pre-registered*

After scanning, participants answered questions about their experience of the study using FormR.^98^ Participants rated responses to the following questions on a scale from 0-10: (i) how well they were able to adopt an efficient *strategy* against each partner, (ii) how *successful* they were against each partner, (iii) how much *fun* it was to play each partner, (iv) how much *sympathy* they had for each partner when they lost, and then each partner’s (v) *competitiveness,* and (v) *intelligence*.

##### Inclusion of others and self (IOS)

The Inclusion of Others and Self (IOS) is a measure of closeness and interconnectedness between two individuals.^99^ A series of 7 increasingly overlapping circles are presented to the participant on paper. Each pair of circles contains the word “self” in one circle and “other” in the other circle. Participants are then asked to choose which circle represents their relationship to the agent in question. We asked participants to show which set of overlapping circles best describes the following agents: (1) computer, (2) mechanoid robot, (3) humanoid robot, (4) a human stranger, (5) the human from the experiment (LEJ), and (6) a close friend. Non-robot items were included for comparison to determine where the robot stood relative to other people in the participant’s lives. The IOS provides another way to address the participant’s view of their relationship to various humans and robots. Responses from the paper and pencil format of the IOS were recorded onto a 7-point scale from 1 (no overlap) to 7 (nearly complete overlap).

### Quantification and statistical analyses

#### fMRI analyses

##### ROI

###### *Pre-registered*

Repeated measures ANOVAs were run for each ROI to assess the effect of game-partner and pairwise comparisons were run only if a main effect of game-partner was found. All pairwise comparisons were corrected for multiple comparisons (Bonferroni). Greenhouse-Geisser corrections were made if any rmANOVA was found to violate Mauchley’s tests of sphericity. We assessed the linear effect of human-likeness using a linear repeated contrast in a within-subject ANOVA, which compares means across the different levels of the independent variable according to the following order: computer < mechanoid < humanoid < human.

###### *Exploratory*

Ratings results from the *Fun*, *Competitiveness*, and *Sympathy* questions in the Debrief, suggested swapping the robot orders in the linear model (see below). As an exploratory analysis, we ran a linear repeated contrast in a within-subject ANOVA to compare means across different levels of the independent variable according to the following order based on socialness ratings: computer < humanoid < mechanoid < human.

Additionally, we assessed whether the pSTS would show a linear pattern based on human-likeness or socialness during game play and whilst watching the video introduction which preceded each round.

##### Whole brain

###### *Pre-registered*

A GLM comprising the four conditions (CP = Computer Partner, MR = Mechanoid Robot, HR= Humanoid Robot, HP = Human Partner) was specified for each participant. Simple contrasts were compared against: (1) HP > CP, (2) HR > CP, (3) MR > CP, (4) HR > MR, (5) HP > HR. Based on previous findings (Krach et al., 2010) and our hypothesis, we expected to see a linear increase in neural activity based on human-likeness of agent. To evaluate this, we calculated a parametric modulation of gameplay partner (actual model weights used: CP = -3, MR = -1, HR = 1, HP = 3). For the second level group analyses, we used a FWE-corrected threshold (p_uncorr_ < 0.001) and a minimum cluster size (k = 100).

###### *Exploratory*

While not pre-registered, we also included the following simple contrasts: (6) HP > MR, (7) MR > HR. We also calculated the parametric modulation of gameplay partners based on socialness (actual model weights used: CP = -3, HR = -1, MR = 1, HP = 3).

#### Behavioral analyses

##### Debrief questions

###### *Pre-registered*

As pre-registered, rmANOVAs were run on each question to assess the effect of agent. Pairwise comparisons between agents were run only if an agent effect was identified. All pairwise comparisons were corrected for multiple comparisons (Bonferroni). Greenhouse-Geisser corrections were made if any rmANOVA was found to violate Mauchley’s tests of sphericity. We assessed the linear effect of human-likeness using a linear repeated contrast in a within-subject ANOVA, which compares means across different levels of the independent variable.

###### *Exploratory*

Furthermore, based on participant-reported perceptions of socialness of the individual agents, we ran an exploratory (not pre-registered) linear repeated contrast in a within-subjects ANOVA that reversed the order of the robots in the 4-element hierarchy within the linear model.

##### Inclusion of others and self (IOS)

###### *Pre-registered*

As pre-registered, rmANOVA was run to assess the effect of agent and pairwise comparisons were run only if an effect of agent was found. All pairwise comparisons were corrected for multiple comparisons (Bonferroni). Greenhouse-Geisser corrections were made if any rmANOVA was found to violate Mauchley’s tests of sphericity.
