# Supplementary material for: Beyond human-likeness: Socialness is more influential when attributing mental states to robots
Source: iScience. 2024 May 22;27(6):110070. doi: 10.1016/j.isci.2024.110070 (PMC11214418; doi:10.1016/j.isci.2024.110070)
Supplement: Document S1. Figures S1–S4 and Tables S1–S6 [file mmc1.pdf]

iScience, Volume *27*

Supplemental information

Beyond human-likeness: Socialness is more influential when attributing mental states to robots

Laura E. Jastrzab, Bishakha Chaudhury, Sarah A. Ashley, Kami Koldewyn, and Emily S. Cross

Supplemental Information

Table S1. T1 acquisition parameters by site, *Related to STAR Methods.*

	Site 1	Site 2
Repetition time (ms)	23	18
Echo time (ms)	3.0	3.5
Acquired voxel size (mm)	1 x 1 x 2	1 x 1 x 2
Reconstructed voxel size (mm)	1 x 1 x 1	1 x 1 x 1
Number of slices (contiguous axial)	176	192
Flip angle	9°	8°
Field of view (mm)	256 x 256	256 x 256
Acquisition matrix size	256 x 220	256 x 220
Reconstruction matrix	256	256
Total acquisition time	6 min	6 min
Pulse sequence	T1	T1
3T MRI system	Siemens TrioTim	Philips Ingenia Elition
Site	University of Glasgow	Bangor University
Note. Both sites used a gradient echo, multi-shot turbo field echo pulse sequence.		

Table S2. BOLD acquisition parameters by site, *Related to STAR Methods.*

	Site 1	Site 2
Repetition time (ms)	2000	2000
Echo time (ms)	13 / 31	12 / 31
Voxel size (mm)	2.75 x 2.75 x 4	2.75 x 2.75 x 4
Number of slices	32	32
Flip angle	85°	85°
Field of view (mm)	220 x 220	220 x 220
Matrix size	80 x 80	80 x 80
Slice acquisition	Ascending	Ascending
Phase encoding	Anterior-posterior	Anterior-posterior
Acquisition orientation	45° from AC-PC	45° from AC-PC
Number of volumes (RPS)*	239	240
Number of volumes (ToM)	174	174
Number of volumes (INT)*	76	72
Pulse sequence	dual-echo EPI	dual-echo EPI
Parallel imaging parameters	GRAPPA accel. factor (3)	SENSE factor (3)
Head coil	32-channel	32-channel
3T MRI system	Siemens TrioTim	Philips Ingenia Elition
Site	University of Glasgow	Bangor University

Note. RPS = Rock Paper Scissors task (main task); ToM = Theory of Mind localizer task (Partly Cloudy movie; Jacoby et al., 2018); INT = Interaction Localizer task (Walbrin & Koldewyn, 2018); Number of volumes for each functional scan were trimmed for analyses so that there were the same number of volumes per site resulting in: (1) 239 volumes for the RPS task, (2) 72 volumes for the Interaction Task (INT).

Table S3. Whole-brain activation peaks in localizers, *Related to STAR Methods.*

		Coordinates			Peak		Cluster-level	
		x	y	z	t-value	k	p_{uncorr}	p_{FWE}
ToM > Pain								
-	Precuneus	16	-56	22	8.30	2524	<0.001	<0.001
R	Temporal-Parietal Junction*	48	-65	32	8.50	1217	<0.001	<0.001
L	Temporal-Parietal Junction	-46	-62	38	6.90	923	<0.001	<0.001
L	Middle Frontal Gyrus	-36	8	54	5.14	145	0.003	0.043
Interaction > Non-Interaction								
R	Posterior Superior Temp Sulcus	54	-48	18	4.92	448	<0.001	<0.001
L	Posterior Superior Temp Sulcus*	-56	-54	22	5.29	158	0.004	0.048
Additional ROIs from other sources					Coordinate Source			
	Dorsal Medial Prefrontal Cortex	-10	56	32	Jacoby et al. (2016)			
	Ventral Medial Prefrontal Cortex	2	54	-12	Jacoby et al. (2016)			
R	Middle Frontal Gyrus	24	28	40	Jacoby et al. (2016)			
R	V1 (BA 17)				WFU PickAtlas			
L	V1 (BA 17)				WFU PickAtlas			

Note. Significance level and size of activation cluster for the primary contrast (both p_{uncorr} and $p_{FWEcorr}$ reported; minimal cluster size, k ≥ 0). Coordinates reported in MNI space. L = Left; R = Right. ToM Localizer uses a short film by Pixar ("Partly Cloudy") to localize mentalizing regions of the brain (Jacoby et al. 2016). As our ToM localizer did not provide mPFC or rmFG coordinates, we created 6mm spheres in Marsbar for those ROIs based on coordinates from Jacoby et al. (2016). rTPJ (true peak coordinates: 50 -68 32) & lpSTS (true peak coordinates: -62 -54 22) coordinates reported in the table above were shifted from true peak so that spheres remained within the brain boundaries.

Table S4. Whole Brain activation peaks with their localization for RPS task, *Related to Figure 2.*

		MNI Coordinates (mm)			Peak t-value	Cluster Extent (voxels)	Cluster-level	
		x	y	z		k	p_{uncorr}	p_{FWE}
**Mechanoid > Computer**								
	-	-	-	-	-	-	ns	ns
**Humanoid > Computer**								
	-	-	-	-	-	-	ns	ns
**Human > Computer**								
-	Medial Prefrontal Cortex	4	48	24	5.95	1264	<0.001	<0.001
R	Temporal-Parietal Junction	50	-46	20	5.42	864	<0.001	<0.001
-	Thalamus	6	8	2	5.23	619	<0.001	0.001
-	Superior Medial Prefrontal Cortex	-2	28	58	5.11	632	<0.001	<0.001
R	Posterior Superior Temporal Sulcus	54	-20	-14	5.07	330	0.001	0.005
	Precuneus	-2	-50	30	4.82	859	<0.001	<0.001
L	Middle Frontal Gyrus	-42	14	50	4.65	318	0.001	0.006
R	Middle Frontal Gyrus	40	12	52	4.58	285	0.001	0.010
L	Temporal-Parietal Junction	-54	-50	26	4.56	390	<0.001	0.002
L	Cerebellum	-14	-82	-32	4.33	252	0.002	0.018
**Human > Humanoid**								
R	Temporal-Parietal Junction	46	-54	28	7.24	1556	<0.001	<0.001
R	Pars Triangularis (IFG)	48	26	18	7.06	3731	<0.001	<0.001
R	Posterior Superior Temporal Sulcus	60	-22	-12	6.37	1238	<0.001	<0.001
-	Precuneus	4	-60	34	5.65	1361	<0.001	<0.001
-	Cerebellum Crus 1	-20	-82	-30	5.18	600	<0.001	<0.001
R	Middle Frontal Gyrus	34	32	44	4.83	1045	<0.001	<0.001
-	Nucleus Accumbens	8	10	-14	4.78	222	0.004	0.031
L	Temporal-Parietal Junction	-48	-48	30	4.39	428	<0.001	0.001

Human > Mechanoid								
R	Temporal-Parietal Junction	48	-44	26	5.20	907	<0.001	<0.001
L	Cerebellum Crus 1	-20	-82	-30	5.18	261	0.002	0.018
R	Precuneus	6	-56	32	4.90	365	<0.001	0.004
R	Posterior Superior Temporal Sulcus	52	-20	-8	4.21	212	0.005	0.039
Humanoid > Mechanoid								
-	-	-	-	-	-	-	-	-
Mechanoid > Humanoid								
	Nucleus Accumbens	-4	10	-10	4.91	313	<0.001	0.003
Human-likeness (CP > MR > HR > HP)								
-	Medial Prefrontal Cortex	2	48	24	5.46	483	<0.001	<0.001
R	Temporal-Parietal Junction	50	-46	22	5.27	778	<0.001	<0.001
-	Superior Medial Prefrontal Cortex	0	32	54	4.72	453	<0.001	0.001
-	Precuneus	12	-50	42	4.63	616	<0.001	<0.001
-	Nucleus Accumbens	6	8	2	4.63	242	0.002	0.018
R	Middle Frontal Gyrus	34	54	4	4.53	188	0.005	0.048
L	Middle Frontal Gyrus	-46	14	44	4.22	225	0.003	0.025
Socialness (CP > HR > MR > HP)								
-	Medial Prefrontal Cortex	-6	44	18	6.12	1727	<0.001	<0.001
R	Temporal-Parietal Junction	50	-50	18	5.52	859	<0.001	<0.001
-	Nucleus Accumbens	6	8	2	5.40	745	<0.001	<0.001
R	Posterior Superior Temporal Sulcus	56	-28	-12	5.30	403	<0.001	0.002
L	Posterior Superior Temporal Sulcus	-56	-22	-10	5.26	242	0.002	0.022
L	Middle Frontal Gyrus	-24	54	18	5.16	270	0.002	0.014
L	Supplementary Motor Area	-2	26	58	5.14	819	<0.001	<0.001
-	Precuneus	12	-52	42	4.86	954	<0.001	<0.001
L	Middle Frontal Gyrus	-42	14	50	4.82	332	0.001	0.006
R	Pars Triangularis	46	24	14	4.74	618	<0.001	<0.001
L	Temporal-Parietal Junction	-58	-50	28	4.64	506	<0.001	<0.001

R	Middle Frontal Gyrus	40	12	52	4.57	407	<0.001	0.002
L	Cerebellum Crus 2	-12	-82	-44	4.45	285	0.001	0.011

Note. Significance level and size of activation cluster for each of the contrasts and the parametric modulation listed above (both p_{uncorr} and $p_{FWEcorr}$ reported; no minimal cluster threshold). Coordinates reported in MNI space.

Table S5. rmANOVAs for PSC in each ROI, *Related to Figure 1.*

		Within-Subjects Effects				Pairwise Comparisons<br>P-value / Cohen's d				Within-Subjects Contrasts (Linear)<br>H: Humanness model<br>S: Socialness model				
	Mean ± SD	*df*	*F*	p	η_p^2	CP	MR	HR	HP		*df*	*F*	p	η_p^2
**rTPJ**[§]														
Computer	-0.15 ± 0.21	2.5, 103.83	6.78	<0.001	0.14		0.09	0.14	-0.14	H	1, 41	3.67	0.063	0.08
Mechanoid	-0.17 ± 0.23					ns		0.04	-0.22					
Humanoid	-0.18 ± 0.23					ns	ns		-0.26	S	1, 41	4.53	0.039	0.10
Human	-0.12 ± 0.23					ns	**	***						
**lTPJ**														
Computer	-0.03 ± 0.23	3, 123	7.60	<0.001	0.16		-0.09	0	-0.37	H	1, 41	7.60	<0.001	0.16
Mechanoid	-0.01 ± 0.21					ns		0.10	-0.29					
Humanoid	-0.03 ± 0.21					ns	ns		-0.39	S	1, 41	12.68	<0.001	0.24
Human	0.05 ± 0.20					***	**	**						
**rmFG**[§]														
Computer	-0.12 ± 0.21	2.56, 104.84	2.28	0.092	0.05									
Mechanoid	-0.14 ± 0.20													
Humanoid	-0.15 ± 0.21													
Human	-0.11 ± 0.19													
**lmFG**[§]														
Computer	0.00 ± 0.17	2.27, 92.92	3.17	0.040	0.07		-0.17	0	-0.28	H	1, 41	4.62	0.038	0.10
Mechanoid	0.03 ± 0.18					ns		0.17	-0.11					
Humanoid	0.00 ± 0.18					ns	ns		-0.27	S	1, 41	7.11	0.011	0.15
Human	0.05 ± 0.19					ns	ns	ns						
**Precuneus**														
Computer	-0.18 ± 0.17	3, 123	1.04	0.376	0.03									
Mechanoid	-0.17 ± 0.19													

Humanoid	-0.19 ± 0.18													
Human	-0.16 ± 0.20													
**dmPFC**														
Computer	-0.22 ± 0.21	3, 123	2.19	0.092	0.05									
Mechanoid	-0.20 ± 0.20													
Humanoid	-0.24 ± 0.22													
Human	-0.20 ± 0.20													
**vmPFC**														
Computer	-0.50 ± 0.44	3, 123	2.16	0.097	0.05									
Mechanoid	-0.49 ± 0.50													
Humanoid	-0.55 ± 0.51													
Human	-0.47 ± 0.43													
**rpSTS**														
Computer	-0.02 ± 0.25	3, 123	12.39	<0.001	0.23		-0.08	0	-0.38	H	1, 41	18.87	<0.001	0.315
Mechanoid	0.00 ± 0.25					ns		0.08	-0.30					
Humanoid	-0.02 ± 0.25					ns	ns		-0.38	S	1,41	25.27	<0.001	0.381
Human	0.07 ± 0.22					***	**	***						
**rpSTS** (Video 1)														
Computer	0.07 ± 0.14	3, 123	29.40	<0.001	0.42		-0.73	-0.54	-1.40	H	1, 38	52.30	<0.001	0.561
Mechanoid	0.20 ± 0.21					***		0.20	-0.57					
Humanoid	0.16 ± 0.19					ns	0.029		-0.80	S	1, 41	77.98	<0.001	0.655
Human	0.32 ± 0.21					***	***	***						
**lpSTS**														
Computer	-0.11 ± 0.26	3, 123	6.96	<0.001	0.15		-0.08	0	0.91	H	1,41	16.33	<0.001	0.285
Mechanoid	-0.09 ± 0.26					ns		0.07	0.98					
Humanoid	-0.11 ± 0.29					ns	ns		0.86	S	1, 41	16.22	<0.001	0.283
Human	-0.35 ± 0.27					***	*	*						
**lpSTS** (Video1)														

Computer	0.04 ± 0.20	3, 123	13.2 6	<0.001	0.24		-0.32	-0.20	-0.79	H	1, 41	22.64	<0.001	0.356
Mechanoid	0.10 ± 0.18					0.056		0.11	-0.50					
Humanoid	0.08 ± 0.20					ns	ns		-0.58	S	1, 41	31.27	<0.001	0.433
Human	0.19 ± 0.18					***	**	***						
**rV1§ (BA17)**														
Computer	0.20 ± 0.39	2.42, 91.93	1.10	0.341	0.03									
Mechanoid	0.20 ± 0.34													
Humanoid	0.19 ± 0.37													
Human	0.25 ± 0.36													
**lV1§ (BA17)**														
Computer	0.09 ± 0.27	2.50, 102.19	0.97	0.400	0.23									
Mechanoid	0.09 ± 0.27													
Humanoid	0.08 ± 0.26													
Human	0.12 ± 0.23													
Note: Total N = 42. All p-values reported are Bonferroni corrected, *p<0.05, **p<0.01, ***p<0.001. §Greenhouse-Geisser correction.														

Table S6. rmANOVAs for Debrief Questions, *Related to Figure 3*

		Within-Subjects Effects				Pairwise Comparisons<br>P-value / Cohen's d				Within-Subjects Contrasts (Linear)<br>H: Humanness<br>S: Socialness				
	**Mean ± SD**	***df***	***F***	**p**	**η_p^2**	**CP**	**MR**	**HR**	**HP**		***df***	***F***	**p**	**η_p^2**
**Successful**														
Computer	6.00 ± 2.04	3, 123	0.50	0.685	0.012	-				-				
Mechanoid	5.83 ± 1.83													
Humanoid	5.52 ± 1.49													
Human	5.76 ± 1.78													
**Strategy**														
Computer	5.24 ± 2.45	3, 123	0.32	0.811	0.008	-				-				
Mechanoid	5.29 ± 1.83													
Humanoid	5.36 ± 1.87													
Human	6.62 ± 1.78													
**Intelligence**§														
Computer	5.21 ± 2.82	2.51, 102.91	12.16	< 0.001	0.229		-0.09	-0.34	-0.92	H	1, 41	25.64	<.001	.385
Mechanoid	5.43 ± 1.96					ns		-0.30	-1.02					
Humanoid	6.05 ± 2.13					ns	ns		-0.69	S	1, 41	14.80	<.001	.265
Human	7.48 ± 2.03					***	***	.007						
**Fun**														
Computer	3.74 ± 2.43	3, 123	33.90	< 0.001	0.453		-1.39	-0.74	-1.75	H	1, 41	60.75	<.001	.597
Mechanoid	7.02 ± 2.30					***		0.64	-0.18					
Humanoid	5.52 ± 2.39					***	.006		-0.91	S	1, 41	85.74	<.001	.677
Human	7.38 ± 1.65					***	ns	***						
**Competitive**														

Computer	3.24 ± 3.03	3, 123	17.24	< 0.001	0.296		-1.16	-0.68	-115	H	1, 41	25.99	<.001	.388
Mechanoid	6.57 ± 2.69					***		0.47	0.15					
Humanoid	5.26 ± 2.87					***	.049		-0.38	S	1, 41	34.36	<.001	.456
Human	6.21 ± 2.68					***	ns	ns						
**Sympathy**§														
Computer	0.71 ± 1.35	2.50, 102.58	58.59	< 0.001	0.588		-1.08	-0.98	-2.87	H	1, 41	136.94	<.001	.770
Mechanoid	3.45 ± 3.32					***		0.28	-1.11					
Humanoid	2.64 ± 2.44					***	ns		-1.63	S	1, 41	149.58	<.001	.785
Human	6.81 ± 2.68					***	***	***						

Note: Total N = 42. All p-values reported are Bonferroni corrected, *p<0.05, **p<0.01, ***p<0.001. §Greenhouse-Geisser correction. Cohen's D for paired-sample t-test: $(x_1 - x_2) / S_{pooled}$ ($S_{pooled} = \sqrt{(S_1^2 - S_2^2) / 2}$).

Figure S1. Whole brain T-map overlap analysis, *Related to Figure 2*.

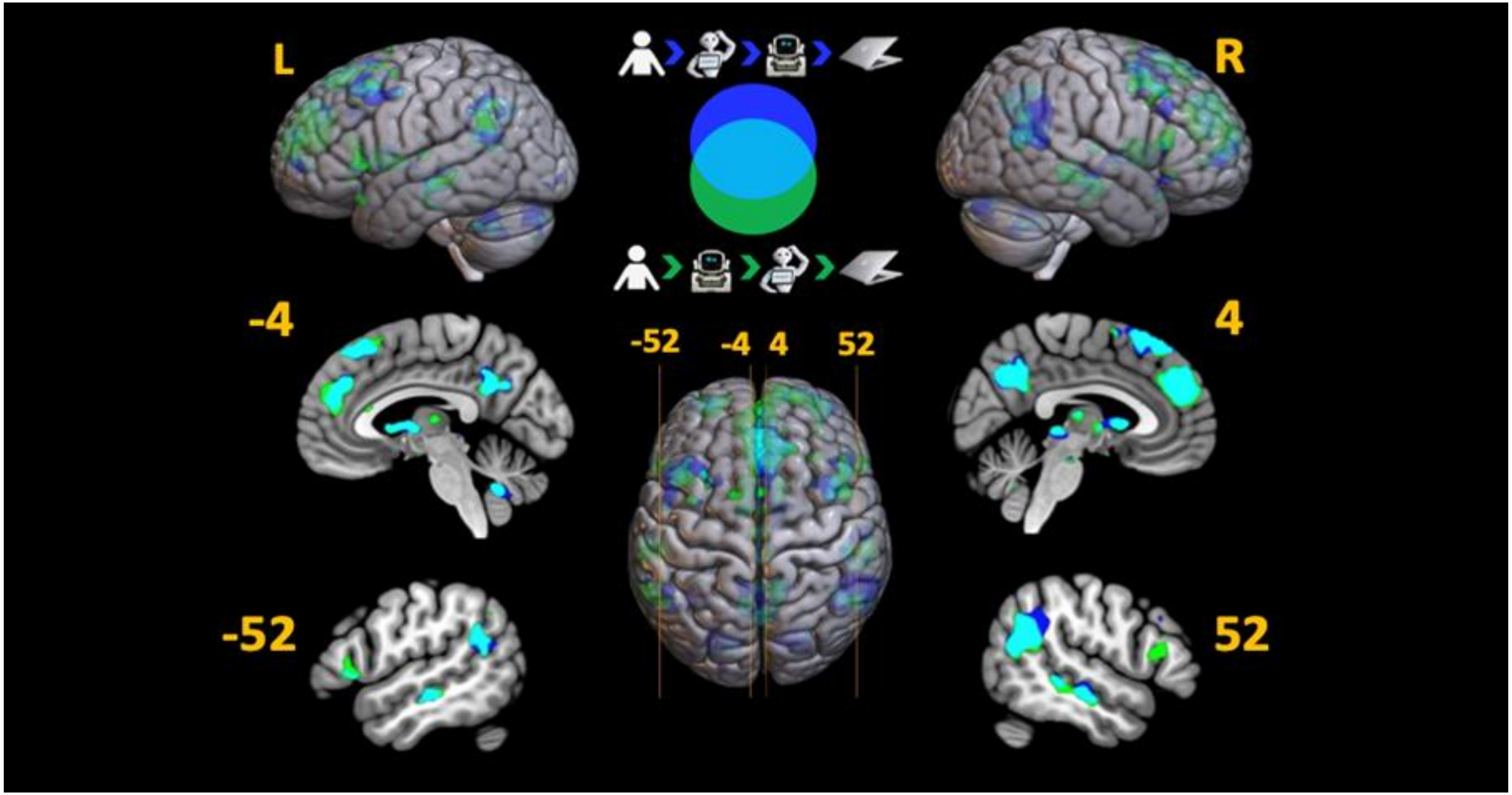

Note. Humanness Model (Human > Humanoid > Mechanoid > Computer ; Blue) and the Socialness model (Human > Mechanoid > Humanoid > Computer; Green) - overlap is Teal. p>0.001 unc, no minimal cluster threshold.

Figure S2. Distribution of percent signal change (PSC) across the 4 game partners in each ROI, *Related to Figure 1*.

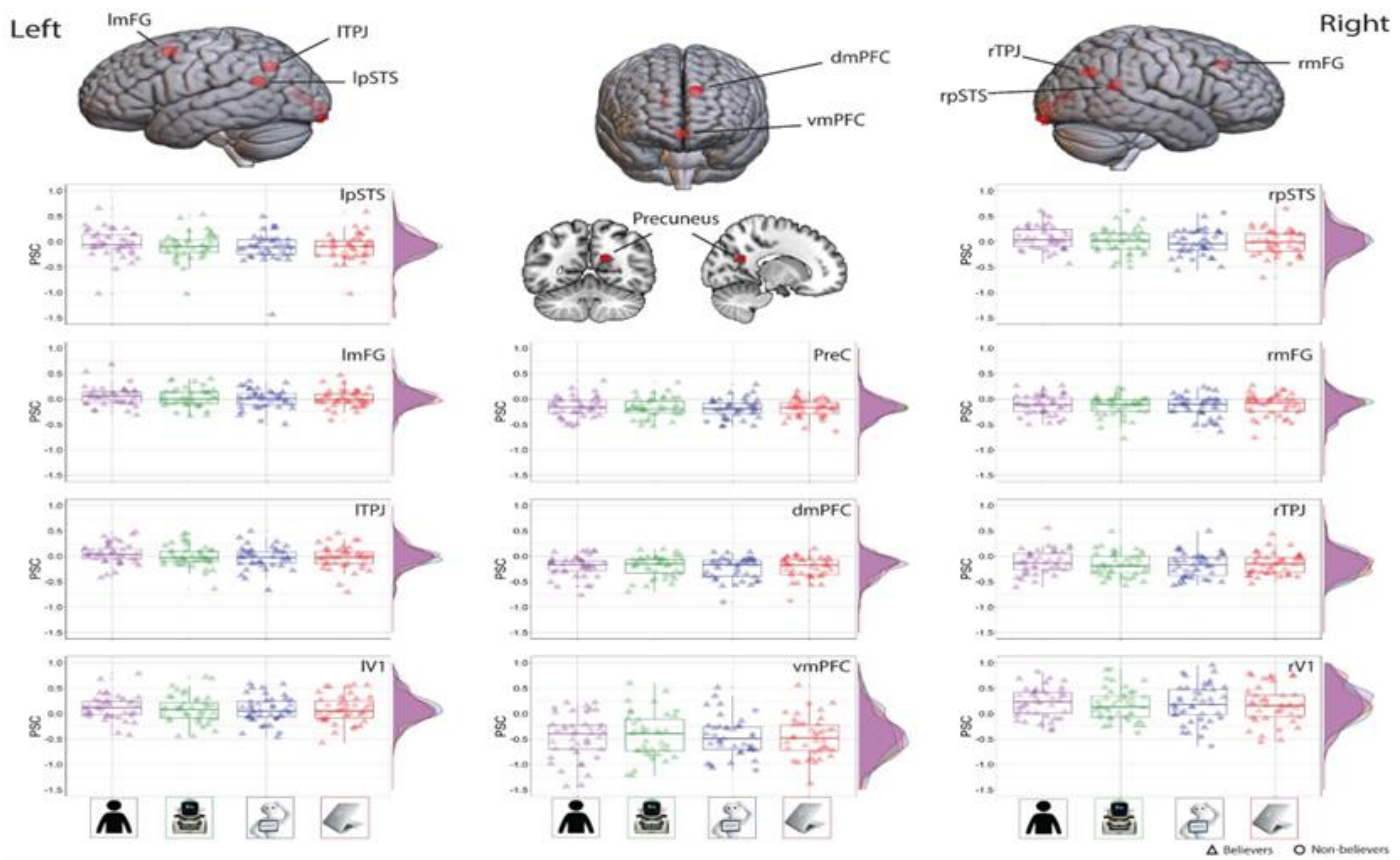

Note. Example 6mm ROI search spheres; see Table S3 for peak coordinates. mFG = left middle frontal gyrus; TPJ = temporal parietal junction; pSTS = posterior superior temporal sulcus; vmPFC = ventromedial prefrontal cortex; dmPFC = dorsomedial prefrontal cortex; PreC = precuneus. Boxplots show 1st-3rd quartiles. Triangular data points represent “true believers”; circular data points represent “non-believers.”

Figure S3. Distribution of rating responses for each of the six debrief questions, *Related to Figure 3.*

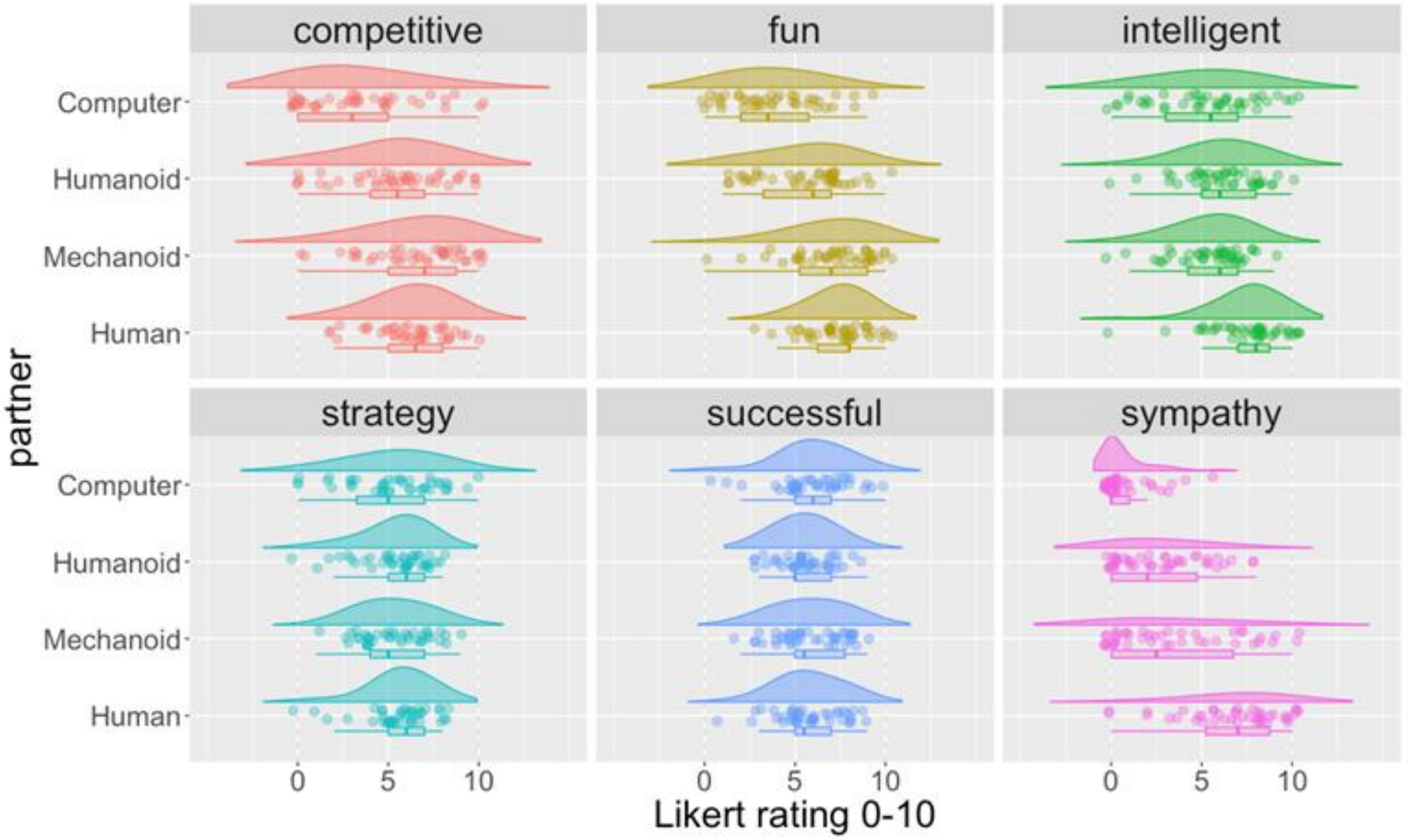

Note. Boxplot show 1st-3rd quartiles.

Figure S4. Experimental paradigm, *Related to STAR Methods.*

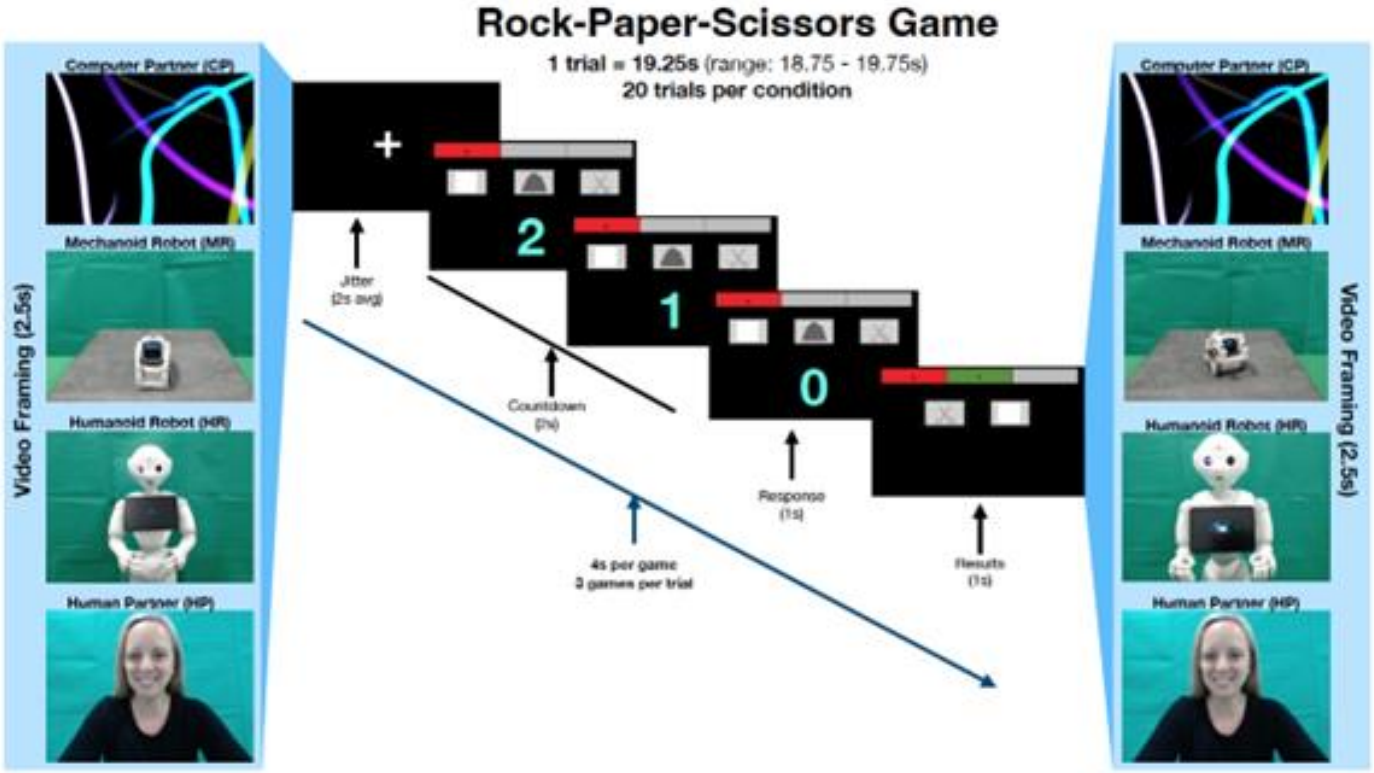

Note. Each game lasts 4 seconds and includes a countdown from 2 to 0; participants respond on ‘0’ and then the results of the game are shown to the participant before the next game in the series commences. Participants play 3 games against one opponent per trial. Each trial is preceded by a 2.5 second video of their opponent (video framing) and concluded by a 2.75 second (video feedback) of their opponent; each series of 3 games lasts 12 seconds without video framing and feedback (average of 19.25 second in total).
